# Biological activity of extracts and hydrolysates from early- and adult-stage edible grasshopper *Sphenarium purpurascens*

**DOI:** 10.3389/fnut.2022.1028543

**Published:** 2022-11-10

**Authors:** M. Selene Marín-Morales, Celeste C. Ibarra-Herrera, Diego A. Luna-Vital, Juan L. Monribot-Villanueva, José A. Guerrero-Analco

**Affiliations:** ^1^NatProLab, Biomolecule Research Lab, Department of Bioengineering, School of Engineering and Science, Tecnológico de Monterrey, Puebla, Mexico; ^2^The Institute for Obesity Research, Tecnológico de Monterrey, Monterrey, Mexico; ^3^Laboratorio de Química de Productos Naturales, Red de Estudios Moleculares Avanzados, Instituto de Ecología A.C., Clúster Científico y Tecnológico BioMimic, Xalapa, Mexico

**Keywords:** edible insects, grasshopper, antioxidant, bioaccessibility, phenolic compounds

## Abstract

Edible insects have become a promising food source because they are rich in protein, fatty acids, minerals, among others. In recent years, edible insects have been proposed to be used as innovative functional ingredients in terms of biological activity. The present study aimed to determine and compare biological activities of the extracts and hydrolysates obtained from early- and adult-stage edible grasshoppers *Sphenarium purpurascens* to evaluate their potential as a source of bioactive compounds. Proximal analyses showed that in adult grasshoppers (AGs), the percentage of protein (48.9% ± 1.2), crude fat (13.1% ± 0.09), and chitin (15.6% ± 0.81) was significantly higher than early grasshoppers (EGs) (42.2% ± 0.55, 9.35% ± 0.08, and 10.5% ± 0.15, respectively). Total phenolic compounds, 2,2-diphenyl-1-picrylhydrazyl (DPPH•), and 2,2′-azinobis-(3-ethylbenzthiazoline-6-sulfonic acid) (ABTS•+) free radical scavenging were analyzed and reported. Enzymatic hydrolysis increased the concentration of total phenolic compounds and higher antioxidant capacity (up to 252.78 mM trolox). Once fractionated by ultrafiltration, the fraction that presented the highest antioxidant activity against DPPH• and ABTS•+ was that with molecules ≤ 10 kDa. Furthermore, the bioaccessibility of the samples was analyzed by *in vitro* protein digestion using a multienzymatic method, and a recovery index (RI) was reported. Extracts and hydrolysates were analyzed by UPLC-MS, and this allowed the identification of phenolic acids and flavonoids. The results obtained in this work suggest that the grasshopper can be used as a possible source of bioactive compounds that can be used in the food or pharmaceutical industry.

## Introduction

Entomophagy is refers to the intake of insects as a source of food; nowadays, entomophagy has increased worldwide as edible insects represent an important source of nutrients such as proteins, fatty acids, vitamins, and minerals (1). Recently, not only the nutritional value of edible insects has been demonstrated by several studies but also antioxidant, anti-inflammatory, antimicrobial, among other bioactivities have been reported (2). The nutrients and biological activities can vary due to several factors, such as diet and environmental conditions in which the insect develops. One of the factors that affect the concentration of nutrients is the morphological stage since it was found that the protein content was higher in the mature stages of insects, and the opposite happened with the fat content, which was found in higher contents in larval stages (3). Also, the taxonomic order of insects influences their proximal composition, for example, the order Orthoptera is richer in protein (61.32 g/100 g dw) than the order Blattodea (35.34 g/100 dw) (4). Despite the great nutritional value of edible insects, the general acceptance of edible insects is low, which has limited their inclusion as part of the daily diet. For this reason, current trends point out the extraction of main nutrients and bioactive molecules to be used as ingredients in food formulations (5). Antioxidant and anti-inflammatory activities from hydrolysates of edible insects have been reported in crickets (*Gryllodes sigillatus*), mealworm larvae (*Tenebrio molitor*), and desert locusts (*Schistocerca gregaria*) (6). Some compounds with antioxidant activity are mainly polyphenols, which provide several benefits to human health. There is evidence that polyphenols can be used as coadjutants in certain diseases such as cardiovascular diseases and inflammatory diseases (7). In this way, polyphenols can be used as natural antioxidants in foods as functional ingredients, thus increasing their nutritional value (8). Phenolic compounds have been reported in edible insects like *Acheta domesticus* (adult) and *T. molitor* (larvae) (9). Even though insects have potential compounds with antioxidant activity, it is important to consider bioaccessibility. It is known that the liberation and solubilization of nutrients and bioactive compounds are affected during digestion (10). Although bioactivities of some insect proteins and hydrolysates have been identified, the number of studies is still quite limited, especially in Mexican edible insects.

*Sphenarium purpurascens* is considered an edible insect endemic to Mexico, and its geographical distribution includes the states of Tlaxcala, Puebla, Guanajuato, Queretaro, Hidalgo, and Mexico. Entomophagy is more common in rural communities since access to other foods of animal origin is limited; thus, insects represent an important nutritional component of daily intake and also a significant economic income for hundreds of families in these areas (11). In the last years, grasshoppers have been studied regarding their chemical composition (12), the effect of diet on their chemical composition (13), and their use as an ingredient in food (14). In this context, antimicrobial and antioxidant capacities of the extracts, hydrolyzed extracts, and hydrolyzed fractions from early- and adult-stage *S. purpurascens* are reported, and *in vitro* protein digestibility was carried out to test bio accessibility of the compounds. Furthermore, UPLC-MS was used for the identification of polyphenols.

## Materials and methods

Grasshopper samples were taken from maize fields in Coronango, Puebla, Mexico. The geographical coordinates are the parallels 19^°^ 06′36″ and 19^°^ 10′42″ of north latitude and the meridians 98^°^ 14′54″ and 98^°^ 19′40″ of western longitude to 2,180 m above sea level. The recollection took place in September 2020 for early grasshopper (EG) and November 2020 for adult grasshopper (AG). Grasshoppers were collected and transported alive, and later, they were cleaned, not purged, and washed with distilled water, and frozen at −80^°^C. The samples were freeze-dried, blended (NutriBullet NBR-0601), and stored at room temperature until use.

Folin–Ciocalteu reagent, gallic acid, 2,2-diphenyl-1-picrylhydrazyl (DPPH•), 2,2′-azinobis-(3-ethylbenzthiazoline-6-sulfonic acid) (ABTS•+), (S)-6-methoxy-2,5,7,8-tetramethylchromane-2-carboxylic acid trolox (Sigma Aldrich), serine endoprotease from *Bacillus licheniformis* 2.4L E.C.3.4.21.14 (Sigma Aldrich), Luria Agar, *Escherichia coli* ATCC 25922, *Staphylococcus aureus* ATCC 25923, *Enterobacter aerogenes* ATCC 13048 *Salmonella* sp., and *Pseudomonas aeruginosa* ATCC 77853 were used in this study. Samples of EG, AG, early grasshopper extract (EGE), adult grasshopper extract (AGE), early grasshopper hydrolysate (EGH), adult grasshopper hydrolysate (AGH), and hydrolyzed fractions were tested in this research.

### Proximal analysis

The proximal analysis of the freeze-dried samples of EG and AG was performed according to AOAC methodologies: ash content 942.05; ethereal extract 945.39; percentage of protein by total nitrogen Kjeldahl 991.20, where factor 5.33 was used to obtain the percentage of protein in the whole grasshopper according to Boulos et al. (15), avoiding overestimation of the protein percentage (15); and percentage humidity of 964.22. The methodology used to determine chitin is described by Acosta-Estrada et al. (1) with slight modifications (16).

### Protein extraction

The samples were added to hexane in a ratio of 1:5 w/v and stirred at 150 rpm at 25^°^C for 27 h. Sedimentation was allowed for 1 h, and the lipid layer was removed by simple decantation. The solid residue was left to dry in an oven at 35^°^C for 72 h to remove hexane. Distilled water was added at a ratio of 1:3 w/v, pH was adjusted to 9.0 by adding 4 M of NaOH, and it was stirred for 2 h. Then, it was filtered, and the filtrate was frozen at −80^°^C and lyophilized. Protein determination using the total nitrogen Kjeldahl method was performed, and the factor converted N was 5.6. This was reported as the most accurate without overestimating the concentration of N (15).

### Enzymatic hydrolysis

The hydrolysis of protein-rich extracts from grasshoppers was performed, as described by Silvestre-De-León et al. (17). In brief, freeze-dried extracts from early- and adult-stage grasshoppers were dissolved with distilled water at 1:20 w/v, pH was adjusted with 0.1 N NaOH to pH 8, and then 0.6% (w/v) of protease from *B. licheniformis* (2.4 U/g) was added. Subsequently, they were incubated in a water bath at a temperature of 60^°^C for 30 min. Inactivation of the enzyme was achieved by incubating at 90^°^C for 15 min. Once the samples were cooled down at room temperature, they were centrifuged at 3,220 *g* for 20 min. The degree of hydrolysis (DH) was determined according to the soluble nitrogen–trichloroacetic acid index (18) as follows: to 1 mL of the hydrolyzed extract, 1 mL of 10% trichloroacetic acid was added, and total nitrogen was determined using total nitrogen Kjeldahl of AOAC 991.20 method. The percentage of DH was calculated using the following equation:


$$\%\mathrm{DH}=\frac{\mathrm{mg}\,\mathrm{of}\,\mathrm{soluble}\,\mathrm{nitrogen}}{\mathrm{mg}\,\mathrm{of}\,\mathrm{total}\,\mathrm{nitrogen}}\times 100$$

The hydrolyzed extracts were frozen and then freeze-dried. The samples were solubilized 1:10 w/w in distilled water, and ultrafiltration was carried out using 10 and 30 kDa molecular weight cutoff membranes to obtain three fractions: fraction 1 (>30 kDa), fraction 2 (10–30 kDa), and fraction 3 (<10 kDa). The fractions were freeze-dried and stored until use.

### Determination of percentage of soluble protein

Soluble protein was measured using Bradford assay (Sigma), with bovine serum albumin (Amresco) as a standard, following the instructions of the supplier. The fractions obtained from ultrafiltration were analyzed using a 96 well plate spectrophotometer (Biotek ELx808, Winooski, VT, USA) at 595 nm.

### Determination of antimicrobial activity

Antimicrobial activity was determined by using the disk diffusion test. The methodology carried out was the one proposed by Wang et al. (19) with slight modifications. First, the strains *E. coli* ATCC 25922, *S. aureus* ATCC 25923, *E. aerogenes* ATCC 13048 *Salmonella* sp., and *P. aeruginosa* ATCC 77853 were inoculated in Luria broth and incubated for 48 h at 35^°^C. Once there was growth (presence of turbidity), the test was performed using Luria agar; paper disks were soaked in the samples for 30 s, then placed on an agar plate, and incubated for 48 h at 35^°^C. As a control, amikacin 1:10 v/v was used. The interpretive criteria were as follows: low susceptible, inhibition zone diameter ≤10 mm; intermediate, 10–14 mm; susceptible, 14–19 mm; and highly susceptible ≥19 mm.

### Determination of total phenolic content

To quantify total phenolic compounds, gallic acid was used as standard (20). From each sample, 100 μL was taken, and 400 μL of distilled water was added, later 1,250 μL of 20% sodium carbonate was added, and then 250 μL of 1 N Folin–Ciocalteu reagent was added. They were left to incubate for 2 h, and then the samples were read in a UV–VIS spectrophotometer Hach DR6000i at 760 nm. The results are expressed as gallic acid equivalent (GAE)/g dry weight.

### DPPH• radical scavenging assay

For the evaluation of the antioxidant capacity of the samples, 2,2-diphenyl-1-picrylhydrazyl (DPPH•) assay was used (21). Stock solutions of DPPH• and 5 μM trolox were prepared. Then, 25 μL of each sample was taken, and 75 μL of the corresponding stock solution was added. They were incubated for 30 min in the dark at room temperature, and then the reading was performed at 517 nm. The results are expressed as millimolar trolox equivalents/g dry weight.

### ABTS•+ radical scavenging assay

Free radical scavenging capacity against 2,2′-azino-bis(3-ethylbenzothiazoline-6-sulphonic acid) (ABTS•+) radical was carried out with a ABTS•+ solution published by Re et al. (22) with slight modifications. ABTS solution was prepared, and then potassium persulfate was added; this solution was allowed to settle for 12–16 h. It was diluted with ethanol to obtain a working solution with an absorbance of 0.7–732 nm. A standard solution of 5 μM trolox diluted in ethanol was used to construct a calibration curve. From each sample, 20 μL was mixed with 2 mL of the stock solution; they were left incubating for 10 min at room temperature in the dark. Later, the reading was carried out at 732 nm. The results are expressed as millimolar trolox equivalents/g dry weight.

### Ferric-reducing antioxidant power assay

The ferric reducing power was determined according to 6 with slight modification. To 960 μL of the sample, 1 mL of 0.2 M phosphate buffer (pH 6.6), and 1 mL of 1% potassium ferricyanide (w/v) were added. The samples were incubated at 50^°^C for 20 min, cooled, and mixed with 1 mL of 10% TCA (v/v). In the next stage, the mix was centrifugated at 3,220 *g* for 10 min. To the supernatant, 1 mL of distilled water and 0.2 mL 0.1% ferric chloride (w/v) were added. After a reaction time of 10 min, the absorbance of the solution was measured at 700 nm.

The reducing power was calculated using the following formula:


$$\mathrm{Reducing}\,\mathrm{power}={\text{A}}_{\text{sample}}-{\text{A}}_{\text{control}}$$

where *A*_control_ is the absorbance of solution without a sample (distilled water instead of the sample) and *A*_sample_ is the absorbance of the solution with a sample. The results are expressed as units of absorbance.

### *In vitro* protein digestibility

*In vitro* protein digestibility was performed using a multienzymatic technique (23), using the enzymes: pancreatic porcine trypsin type IX-S (T4799, Sigma Aldrich, St. Louis, MO, USA), α-chymotrypsin type II from bovine pancreas (C4129, Sigma Aldrich, St. Louis, MO, USA); and *Streptomyces griseus* protease type XIV (P5147, Sigma Aldrich, St. Louis, MO, USA). The samples were adjusted to pH 8, and the temperature applied was 37^°^C. The percentage of protein digestibility (% PD) was calculated with the following formula:


$$\%\text{PD}=\frac{\mathrm{Protein}\,\mathrm{content}\,\mathrm{in}\,\mathrm{digested}\,\mathrm{sample}}{\mathrm{Protein}\,\mathrm{content}\,\mathrm{in}\,\mathrm{undigested}\,\mathrm{sample}}\times 100$$

### Recovery index

To analyze the effect of *in vitro* digestion on the concentration of total phenolic compounds and their antioxidant activity, the percentage of the recovery index (RI) was obtained (24). The RI indicates the proportion of phenolic compounds present in the samples after *in vitro* protein digestion according to the following equation:


$$\mathrm{Recovery}\,\mathrm{index}\,\left(\mathrm{RI}\%\right)=\frac{{\text{BC}}_{\text{DS}}}{{\text{BC}}_{\text{BD}}}\times 100$$

where BC_DS_ is the bioactives contained in the digested sample and BC_BD_ is the bioactive content quantified in the sample before digestion.

### Identification and quantification of phenolic compounds

Identification and quantification of phenolic compounds were performed as previously reported by Juárez-Trujillo et al. (25) and Monribot et al. (26). The samples were diluted in methanol 1:10 (w/v), filtered with 0.2 μm PTFE membranes, and placed in 2 mL UPLC vials. Chromatographic and spectrometric conditions: Data were obtained with a 1290 Infinity Agilent ultra-high resolution liquid chromatograph coupled to a 6460 Agilent triple quadrupole mass spectrometer using an Agilent, Eclipse Plus C18, 2.1 × 50 mm, 1.8 microns column. The mobile phases were water with 0.1% of formic acid (A) and acetonitrile with 0.1% formic acid (B), both in MS grade. The gradient starts with 1% of B, then changed to 50% of B in 30 min. After that, the gradient changed to 99% of B in 5 min followed by an isocratic step for 4 min at 99% of B. Finally, the gradient changed to 1% of B in 1 min, followed by an isocratic step for 5 min. The flow was 0.3 mL/min, and 2 μL of samples were injected. The column oven temperature was 40^°^C. The gas temperature and flow were 300^°^C and 5 L/min, respectively. The sheath gas temperature and flow were 250^°^C and 11 L/min, respectively. The nebulizer pressure was 45 psi. The capillary and nozzle voltages were 3,500 and 500 V, respectively. The dynamic Multiple Reaction Monitoring (dMRM) transitions, collision energy and fragmentor voltages, and determination coefficients for each identified compound are shown in Supplementary Table 1.

### Statistical analyses

All the samples were analyzed in triplicate, the standard deviation was calculated, and Anderson–Darling normality test was used to analyze data distribution. The data obtained from proximal analyses were compared between the samples by using *post-hoc* Student’s *t*-test using Minitab^®^ 19 software (State College, PA, USA). For the profile of polyphenols and percent of soluble protein, data obtained were statistically analyzed using one-way analysis of variance (ANOVA) and Tukey test, using Minitab^^®^^ 19 software from Minitab Inc., (State College, PA, USA). The values were considered statistically different when *p*-values were lower than 0.05.

## Results and discussion

### Proximal composition

The chemical characterization of EG and AG is presented in Table 1. Regarding protein content in EG, 42.2% ± 0.55, and in AG, 48.9% ± 1.2, a statistical difference was found (*p*-value < 0.05). These results are in the same range of 40–70% that previous works reported for these species (13, 27). Compared with similar edible insects as black cricket *Gryllus assimilis* (36% of protein) (28), both stages of grasshoppers contained a higher quantity of protein. *Schistocerca piceifrons* contains 80.2% of protein, fat 6.2%, ashes 3.3%, and chitin 11.8% (29), where the content of protein is higher than the value found in grasshoppers. In the case of fat content, 9.35% ± 0.08 was obtained in EG and 13.1% ± 0.09 in AG, which are higher than the fat content reported in *S. piceifrons*. *G. assimilis*, and *S. piceifrons* belong to the same order Orthoptera as *S. purpurascens* and have similar morphological characteristics. Other Mexican edible insects such as *Aegiale hesperiaris* (white worm), *Comadia redtenbacheri* (red worm), and *Liometopum apiculatum* (escamol) presented 37.79, 31.23, and 36.98% of protein, respectively, which are lower than those obtained in this work (30). Compared with EG and AG samples, statistical differences (*p*-value < 0.05) were found. AG presented a higher content of fat (13.1% ± 0.09), protein (48.9% ± 1.2), and chitin (15.6% ± 0.81) and a lower content of ashes than EG. Therefore, the proximal content of the grasshopper is affected by several factors, in this case, the stage of development. This has been previously reported (3). The content of protein in EGE is 50.6% ± 1.1, and that in AGE is 54.4% ± 0.14, which increased by 20.0 and 11.3%, respectively, compared with those in the whole insect. The protein content in the extract of *S. gregaria* increased by 14.8% compared with the protein content in the whole insect (31).

**TABLE 1:** Proximal composition of grasshopper samples in early and adult stages.

Parameters	Early grasshopper (EG)	Adult grasshopper (AG)
Ash	11.5^a^ ± 0.32	7.94^b^ ± 0.13
Ethereal extract	9.35^a^ ± 0.08	13.1^b^ ± 0.09
Carbohydrates	16.1^a^± 0.09	11.8^b^± 0.05
Protein	42.2^a^ ± 0.55	48.9^b^ ± 1.2
Chitin	10.5^a^ ± 0.15	15.6^b^ ± 0.81

Data are expressed in *g*/100 *g* of dry matter. All values represent the mean ± SD by triplicate. ^a,b^Different letters between columns indicate a statistically significant difference; *p*-value < 0.05 according to student’s *t*-test.

### Antimicrobial activity

Samples of EGE, AGE, EGH, AGH, and fractions of the hydrolysates did not present an inhibition halo, indicating that there is no presence of compounds with antimicrobial activity. Antimicrobial peptides/polypeptides (AMP) are an innate component of immunity to insects found in their hemolymph and have important biological activity against fungi, viruses, parasites, and, most importantly, antibiotic-resistant bacteria. In some cases, the production of these compounds must be stimulated. For example, in a previous study, housefly larvae were inoculated with a suspension of *Salmonella pullorum* cells to obtain AMP (19). Another example is a study where crickets were infected with *Photorhabdus asymbiotica* and derived from the infection, and glidobactin A, luminmycin, and luminmycin D were isolated (32). Considering that the crickets and grasshoppers belong to the Orthoptera order, it could be possible to obtain antimicrobial compounds stimulating their production.

### Degree of hydrolysis

The DH for EGH was 12.6% ± 0.64 and 13.1% ± 0.6 for AGH, without statistical differences (*p*-value < 0.05). Purschke et al. (33) reported the hydrolysis of flour of *Locusta migratoria* using Alcalase^®^ at 0.05, 0.5, and 1% E:S during 30 min, obtaining 7.3, 9.5, and 11.6% of DH, respectively. In this case, the results of DH obtained in EGH and AGH were higher. Also, an extract of *G. sigillatus* was treated with Alcalase^®^, and a similar DH (15.2%) was obtained; the conditions were 0.25% E:S during 10 min to 50^°^C, pH 8 (34). The degree of hydrolysis depends on certain conditions such as temperature, time, enzymes, and origin of proteins (35). Due to hydrolysis, some molecular properties of proteins change, producing a decrease in molecular weight, release or exposure of hydrophobic groups, among other phenomena (33). As a result of the molecular changes of these molecules, their functional properties are also affected. In this case, the hydrolysates, EGH and AGH, were fractionated and analyzed regarding their antioxidant capacity.

### Fractionation of hydrolysates

In total, three fractions were obtained after ultrafiltration using 30 and 10 kDa molecular weight cutoff membranes: fraction 1 (>30 kDa), fraction 2 (10–30 kDa), and fraction 3 (<10 kDa). In terms of protein, as given in Table 2, fractions 1 and 3 had a higher amount of protein than fractions 2 for both samples EHG and AGH (*p*-value < 0.05). This means that peptides below 10 kDa were obtained after hydrolysis, and an important portion of proteins remained partially hydrolyzed or non-hydrolyzed above 30 kDa (fraction 1). Furthermore, statistical differences (*p*-value < 0.05) were found between all the fractions.

**TABLE 2:** Protein distribution in hydrolyzed fractions from early grasshopper hydrolysates (EGH) and adult grasshopper hydrolysates (AGH).

Sample	EGH protein (%)	AGH protein (%)
Fraction 1 (>30 kDa)	33.01^c^ ± 0.03	43.17^a^ ± 0.04
Fraction 2 (10–30 kDa)	18.82^e^ ± 0.05	13.52^f^ ± 0.03
Fraction 3 (<10 kDa)	32.16^d^ ± 0.03	39.41^b^ ± 0.03

All values are means ± SD by triplicate. ^a,b,c,d,e,f^Different letters between columns indicate a statistically significant difference based on the Tukey test.

### Total phenolic content

**FIGURE 1:**
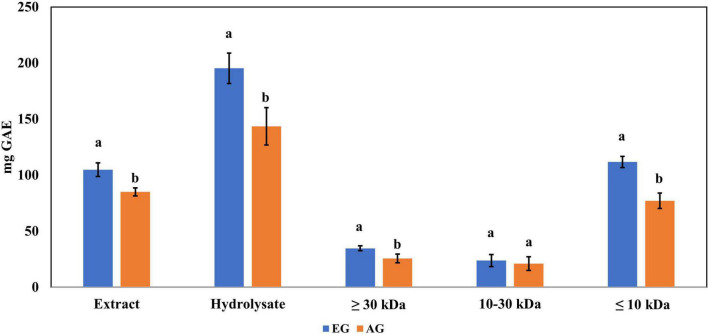
Total phenolic content in extracts, hydrolysates, and hydrolyzed fractions of early grasshopper (EG) and adult grasshopper (AG). Results obtained are reported in milligrams of gallic acid equivalents (mg GAE)/gram dry weight. Different letters between bars indicate significant difference (*p*-value < 0.05).

The enzymatic hydrolysis proved to be an efficient process for releasing compounds with higher biological activity than the non-hydrolyzed samples. The samples of EGE, AGE, EGH, AGH, and hydrolyzed fractions obtained from EGH and AGH (fraction 1: >30 kDa, fraction 2: 10–30 kDa, fraction 3: <10 kDa) were tested. EGH (195.3 ± 13.6 mg GAE) and AGH (143.5 ± 16.7 mg GAE) raised 1.9 and 1.7 times, respectively, in comparison to EGE (104.8 ± 6.1 mg GAE) and AGE (84.98 ± 3.6 mg GAE) (see Figure 1). In addition, the hydrolyzed fraction that obtained the highest content of phenolic compounds was fraction 3 (<10 kDa), where 111.7 ± 4.89 mg GAE was obtained in EGH, and 77.02 ± 6.94 mg GAE in AGH, with statistical difference (*p*-value < 0.05). Some phenolic compounds are linked to macromolecules as proteins, and when proteins are hydrolyzed by enzymes, the release or exposure of such compounds can be given, increasing their content in fractions of low molecular weight (36, 37). The content of total phenolic compounds can be compared with that of aqueous extract from *Henicus whellani* and *Macrotermes facilger* that reported 7.7 and 9.37 mg GAE/dry base 100 *g*, respectively (38); in the aqueous extract of *S. purpurascens*, 12.33 ± 0.54 and 10.92 ± 0.36 mg GAE, in EG and AG samples, were obtained. The results reported here can be compared with other Mexican edible insects like *Ascra cordifera* (jumiles), *Brachygastra mellifica* (wasp), and *Hermetia illucens* that presented 12.8, 10.9, and 3.9 mg GAE of phenolic compounds, respectively (39). It is known that plants are excellent sources of phenolic compounds; for example, plants used to make infusions such as chamomile presented 69.28 mg GAE, and lemon, 71.69 mg GAE, which are similar to those obtained in EGE (104.8 ± 6.1 mg GAE) and AGE (84.98 ± 3.6 mg GAE). On the other hand, the phenolic compound content from plants like spearmint (231.85 mg GAE), arnica (173.3 mg GAE), and boldo (312.71 mg GAE) (40) contains similar amounts as obtained in EGH (195.3 ± 13.6 mg GAE) and AGH (143.5 ± 16.7 mg GAE). In Figure 1, a significant difference (*p*-value < 0.05) can be observed between EG and AG in all the samples, extract, hydrolysates, and fractions. EG samples showed up to 1.4 times higher content of total phenolic compounds. This demonstrates that the content of phenolic compounds can be affected by the stage of the insect.

### Antioxidant activity against DPPH•, ABTS•+, and FRAP

**FIGURE 2:**
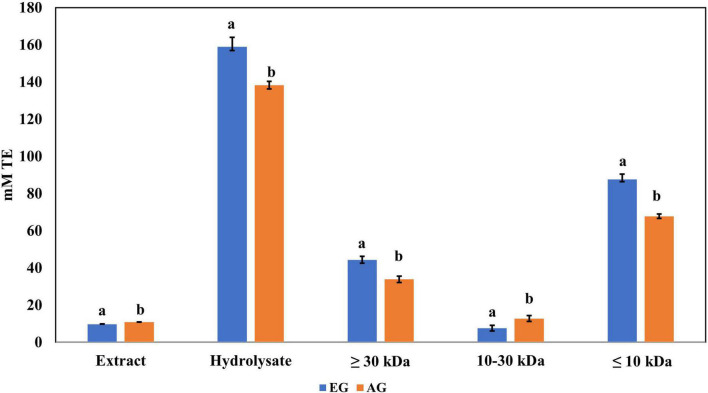
Antioxidant activity against 2,2-diphenyl-1-picrylhydrazyl (DPPH) radical of extracts, hydrolysates, and hydrolyzed fractions of early grasshopper (EG) and adult grasshopper (AG). Results are expressed in millimolar trolox equivalents (mM TE)/gram dry weight. Different letters between bars indicate a significant difference (*p*-value < 0.05).

The samples of EGE, AGE, EGH, AGH, and hydrolyzed fractions obtained from EGH and AGH (fraction 1: >30 kDa, fraction 2: 10–30 kDa, fraction 3: <10 kDa) were tested. EGH (158.9 ± 5.1 mM trolox) and AGH (138.3 ± 2.6 mM trolox) had higher antioxidant activity against DPPH• than EGE (9.75 ± 0.90 mM trolox) and AGE (10.9 ± 0.36 mM trolox), as shown in Figure 2. The content of compounds with antioxidant activity against DPPH• found in EGH and AGH had a considerable increase up to 15 times in comparison with EGE and AGE. Hydrolyzed fractions 3 (<10 kDa) presented higher antioxidant activity (87.56 ± mM trolox in EG and 67.76 ± mM trolox in AG) than in the other fractions (1 and 2) analyzed. In the work carried out by Miranda de Matos et al. (28), fractions with a molecular weight lower than 10 and 5 kDa were also responsible for the highest biological activity for DPPH• and ABTS•+ radical scavenging activities. Other edible insects have shown antioxidant activity against these radicals; for instance, the flour of the insect *Rhynchophorus ferrugineus* presented 2.03 mM trolox against DPPH• in aqueous extract (41). In this study, *S. purpurascens* presented 7.65 ± 0.21 mM trolox and 9.22 ± 0.13 mM trolox in EG and AG samples, respectively.

**FIGURE 3:**
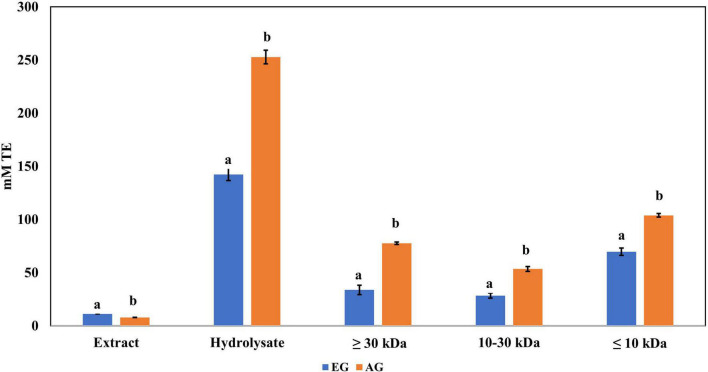
Antioxidant activity against 2,2′-azino-bis(3-ethylbenzothiazoline-6-sulphonic acid) (ABTS) radical of extracts, hydrolysates, and hydrolyzed fractions of early grasshopper (EG) and adult grasshopper (AG). Results obtained are reported in millimolar trolox equivalents (mM TE)/gram dry weight. Different letters between bars indicate significant difference (*p*-value < 0.05).

Regarding ABTS•+ radical scavenging, the results obtained are shown in Figure 3. EGH (142.5 ± 5.9 mM trolox) had a 10-fold scavenging activity compared with EGE (11.22 ± 0.01 mM trolox), and AGH (252.7 ± 6.32 mM trolox) presented 30 times more effectiveness than AGE (7.90 ± 0.16 mM trolox). The values reported corresponding to EGH, and AGH showed 1.5 and 2.7 times higher values than *Alphitobius diaperinus* hydrolysates, which had 95.0 mM trolox against ABTS•+ (42). The soluble extracts of some insects such as grasshoppers (2.55 ± 0.05 mM trolox), silkworms (2.48 ± 0.19 mM trolox), and crickets (2.37 ± 0.03 mM trolox) have shown antioxidant capacity vs. ABTS•+ radical (41), although EG (3.77 ± 0.07 mM trolox) and AG (5.73 ± 0.14 mM trolox) presented higher antioxidant capacity and up to 5 times higher antioxidant capacity than fresh orange juice (0.40 ± 0.01 mM trolox). Furthermore, the fractions 3 (<10 kDa) of AGH and EGH presented higher ABTS•+ radical scavenging (103.9 ± 1.68 and 69.73 ± 3.39 mM trolox) than other fractions.

**FIGURE 4:**
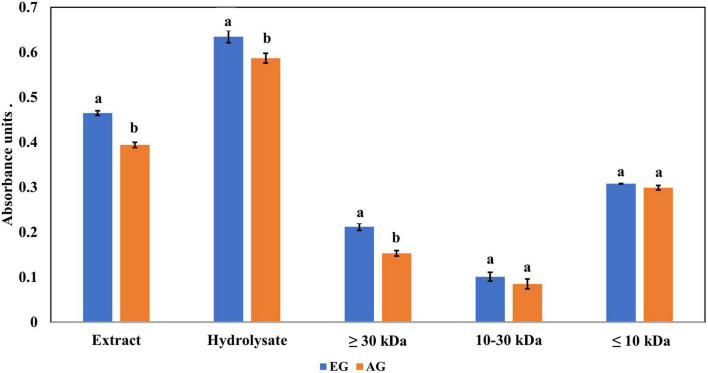
Ferric reducing antioxidant power of extracts, hydrolysates, and hydrolyzed fractions of early grasshopper (EG) and adult grasshopper (AG). Results are reported in absorbance units at 700 nm. Different letters between bars indicate a significant difference (*p*-value < 0.05).

The ferric reducing antioxidant power (FRAP) values are presented in Figure 4, where EGE had 0.465 abs and AGE had 0.394 abs. While EGH had 0.635 abs, AGH had 0.587 abs. Similar results have been found in edible insects such as the hydrolysate of *Amphiacusta annulipes* (0.652 abs), *Zophobas morio* (0.522 abs), and *Gromphadorhina portentosa* (0.485 abs) (35). In phaseolin, the hydrolysate presented 0.062 abs (43), approximately 10 times less than hydrolysates from edible insects. The evaluation of each hydrolyzed fraction showed that fractions 3 (<10 kDa) of EGH (0.308 abs) and AGH (0.299 abs) had higher absorbances than values obtained in fractions 2 (10–30 kDa), EGH (0.101 abs) and AGH (0.086 abs), and fractions 1 (>30 kDa) EGH (0.212 abs) and AGH (0.153 abs). Even though there is a significant difference between EGH and AGH fractions, in the case of fraction 3, there is no significant difference (*p*-value < 0.05).

Antioxidant compounds can be classified into primary antioxidants, which have the ability to inhibit oxidation reactions by the transfer of or donate an electron, and secondary antioxidants, which prevent oxidation by chelation of metal (44). Edible insect hydrolysates contain compounds that can be considered electron donors or receptors and counteract the oxidative effect of radicals. The differences found in antioxidant activities can be mainly related to the chemical structures of proteins, peptides, and other compounds released. For example, it is known that sulfur amino acids are those with the highest radical scavenging capacity (45). In addition, the samples presented significant differences (*p*-value < 0.05), indicating that the stage of the insect has an effect in antioxidant activity against DPPH•, ABTS•+, and ferric reducing antioxidant power.

### *In vitro* protein digestibility and RI

The samples of EG, AG, EGE, AGE, EGH, and AGH were tested. In terms of digestibility, the results were as follows: EG (81.59%^c^ ± 0.13) and AG (85.21%^b^ ± 0.94), with the latter similar to that reported by Ibarra-Herrera et al. (13) for grasshopper fed with maize (87.9%). For EGH (90.82%^a^ ± 0.41) and AGH (92.45%^a^ ± 0.65), a higher digestibility was found than in EG, AG, EGE (83.22%^c^± 0.81), and AGE (86.11%^b^± 0.11). This is related to the availability of proteins and peptides in hydrolysates, which facilitates their digestion (37). There are significant differences (*p*-value < 0.05) between samples of EG and AG, and EGE and AGE, indicating that the stage of the insect affects *in vitro* protein digestibility.

**FIGURE 5:**
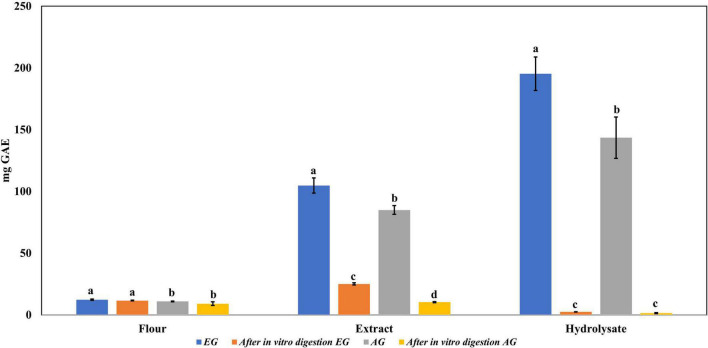
Total phenolic content in flour, extracts, and hydrolysates of early grasshopper (EG) and adult grasshopper (AG) before and after *in vitro* protein digestion. Results obtained are reported in milligrams of gallic acid equivalents (mg GAE)/gram dry weight. Different letters between bars indicate significant difference (*p*-value < 0.05).

**FIGURE 6:**
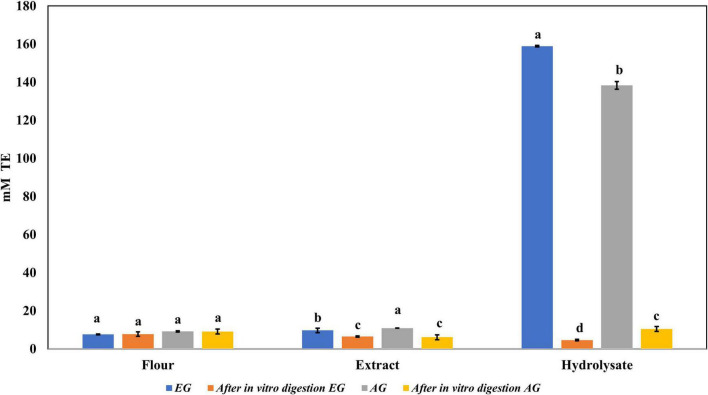
Antioxidant activity against 2,2-diphenyl-1-picrylhydrazyl (DPPH) of flour, extracts, and hydrolysates of early grasshopper (EG) and adult grasshopper (AG) before and after *in vitro* protein digestion. Results are expressed in millimolar trolox equivalents (mM TE)/gram dry weight. Different letters between bars indicate a significant difference (*p*-value < 0.05).

**FIGURE 7:**
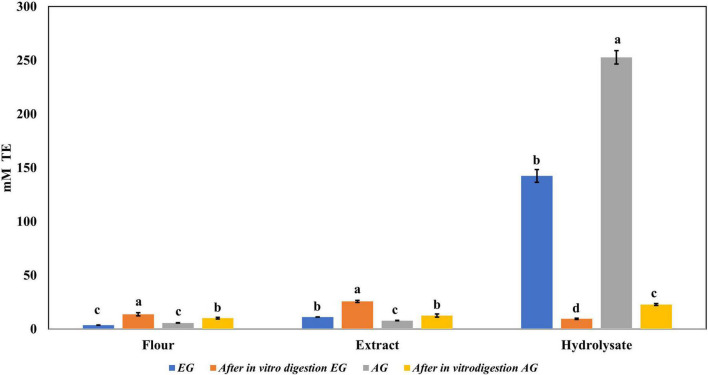
Antioxidant activity against 2,2′-azino-bis(3-ethylbenzothiazoline-6-sulphonic acid) (ABTS) radical of flour, extracts, and hydrolysates of early grasshopper (EG) and adult grasshopper (AG) before and after *in vitro* protein digestion. Results obtained are reported in millimolar trolox equivalents (mM TE)/gram dry weight. Different letters between bars indicate significant difference (*p*-value < 0.05).

**FIGURE 8:**
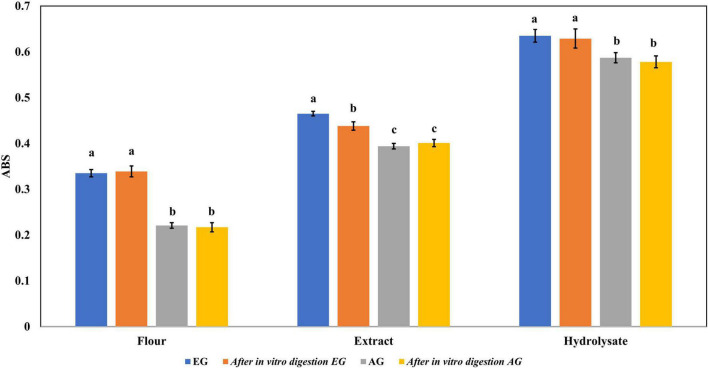
Ferric reducing antioxidant power of flour, extracts, and hydrolysates of early grasshopper (EG) and adult grasshopper (AG) comparing before and after *in vitro* protein digestion. Results are reported in absorbance units at 700 nm. Different letters between bars indicate a significant difference (*p*-value < 0.05).

Regarding the content of total phenolic compounds, it was observed that after *in vitro* protein digestion (Figure 5), the content of phenolics in EGH and AGH decreased drastically (2.51 ± 0.15 and 1.44 ± 0.39 mg GAE, respectively), resulting in an RI of 1.29 and 1.01%, respectively. This indicates that compounds responsible for reducing activity were subjected to enzymatic degradation, which results in a low bioaccessibility of the initial compounds that presented reducing capacity. Despite of this decrease, samples of EGE (25.09 ± 0.86 mg GAE) and AGE (10.39 ± 0.44 mg GAE) still retain some reducing capacity, resulting in an RI around 23.95 and 12.36%, respectively. Reducing capacity of compounds in the samples EG and AG was the highest after *in vitro* protein digestion (RI of 94.40 and 83.50%, respectively). This result is attributed to the protective role of the food matrix preventing from an extensive enzymatic degradation (this is confirmed with low digestibility, around 81–85%, obtained for these samples), confirming that compounds with reducing capacity are not degraded. Similar results were observed in scavenging radical activity for DPPH• (see Figure 6), with a decrease in the samples of EGE (6.53 ± 0.35 mM trolox, RI 66.97%), AGE (6.16 ± 0.37 mM trolox, RI of 56.31%), EGH (4.65 ± 0.35 mM trolox, RI of 2.93%), and AGH (10.52 ± 0.65 mM trolox, RI of 7.61%) after *in vitro* protein digestion. This indicates that compounds present in these samples are degraded into compounds with less antioxidant activity (46). In this case, a low bioaccessibility of compounds with scavenging radical activity for DPPH• is observed. On the contrary, ABTS•+ showed an increase after *in vitro* protein digestion with higher concentrations for EG (13.7 ± 1.4 mM trolox, RI of 365.2%) and AG (10.13 ± 0.80 mM trolox, RI of 176.5%), increasing bioaccessibility of these compounds. In this case, enzymatic digestion degraded the food matrix, allowing the release of compounds with scavenging capacity against ABTS•+ (Figure 7). Similar results for EGE (25.75 ± 1.03 mM trolox, 229.5%) and AGE (12.59 ± 1.5 mM trolox, RI of 150.37%) samples were obtained where enzymatic digestion increased the release of compounds with scavenging capacity against ABTS•+. In the case of hydrolysates, EGH (9.6 ± 0.58 mM trolox, RI of 6.74%) and AGH (22.89 ± 0.87 mM trolox, RI of 9.06%), the enzymatic digestion decreased this effect in the corresponding samples. Although these samples initially had compounds with higher antioxidant capacity, they are mostly exposed to enzymatic degradation, decreasing their bioaccessibility (46). Regarding ferric reducing power, the results obtained (Figure 8) did not present statistical differences (*p*-value < 0.05), demonstrating that enzymatic digestion did neither affect their reducing power nor increase their bioaccessibility. Studies carried out on quinoa seeds showed that acid digestion (RI between 87.6 and 116.7%) and intestinal digestion (RI of 89.6–124%), which help the degradation of the food matrix, exposing certain phenolic compounds (47), as observed for the flour samples of EG and AG in this work. Even though the extracts and hydrolysates of *S. purpurascens* represent a good source of antioxidants, their use as a functional food is diminished by *in vitro* digestion.

### Identification and quantification of total phenolic compounds

In this analysis, the EGE, AGE, EGH, and AGH were tested. UPLC-MS analyses allowed the identification of phenolic compounds. In Table 3 the phenolic compounds found in the samples of EGE, AGE, EGH, and AGH are listed. A different profile was found for all the samples (*p*-value < 0.05), demonstrating that the phenolic compounds can be affected by the stage of the insect and enzymatic hydrolysis of the samples. Most of the phenolic compounds belong to the phenolic acids subgroup, where protocatechuic acid had a high concentration in EGE (114.03 ± 5.9 μg/*g*) and AGE (88.44 ± 9.9 μg/*g*) with significant differences (*p*-value < 0.05). Protocatechuic acid is found in plants such as olives, grapes, and green tea and has been shown to suppress the expression of tumor necrosis factor-alpha (TNF-α), interleukin 1beta (IL-1β), inducible nitric oxide synthase (iNOS), and cyclooxygenase-2 (COX-2) by modulating the NF-κB and MAPK pathways, closely related to inflammatory processes (48). In *S. purpurascens*, phenolic acid could be related to the sclerotization process since this compound has also been found in cockroaches and mantis ootheca, which could provide some rigidity depending on development (36). Also, 4-hydroxybenzoic acid (HBA) was found in a high concentration in EGE (82.15 μg/*g*), 5.8 times higher than that in the rest of the samples. According to Winter et al. (49), HBA is found to decrease oxidative stress by hydrogenous peroxide granule neurons of the cerebellum, which can be attributed to its neuroprotective properties (49). On the other hand, gentisic acid was only found in AGE (12.99 ± 0.05 μg/*g*), and AGH (12.87 ± 0.76 μg/*g*), and this polyphenol is associated with anti-inflammatory activity which has the ability to inhibit low-density lipoprotein oxidation in human plasma (50). In comparison to other Mexican edible insects such as *A. cordifera* and *B. mellifica*, where caffeic acid is found in concentrations of 0.534 mg/*g* and 0.401 mg/*g*, respectively (39), in extracts of grasshoppers, 1.77 ± 0.14 μg/*g* and 7.46 ± 0.05 μg/*g* were obtained. Regardless of the difference between the insects tested, the extraction processes used could also generate differences in the profiles. Within the phenolic aldehyde group, the only one that was quantified was vanillin in the AGE (60.1 ± 0.13 μg/*g*), which is present in some plants and mainly in vanilla. This compound has shown anticancer activity in color cancer cells (51). A coumarin umbelliferone was found in the AGE (0.83 ± 0.01 μg/*g*) and AGH (0.41 ± 0.03 μg/*g*) samples. Umbelliferone is known as a pharmacological agent and is used as a sun protection agent due to its antioxidant capacity (52). Flavonoids in the samples of EG and AG were luteolin, apigenin, quercetin, kaempferol, quercetin-3-glucoside, and kaempferol-3-O-glucoside. Flavonoids have been reported in insects such as *A. domesticus* including quercetin-3-glucoside, quercetin-3-rutinoside, kaempferol-3-glucoside, daidzein, quercetin, naringenin, and apigenin (53). The presence of ferulic acid, *p*-coumaric acid, quercetin and kaempferol could derive from the diet of grasshoppers which mainly consisted in corn leaves (54, 55). On the other hand, non-dietary polyphenols such as coumarin and catechol can be present in the cuticle of insects, due to phenol oxidases, which play an important role in the cuticle structure in a process called sclerotization (36).

**TABLE 3:** Identification and quantification of phenolic compounds in early grasshopper extract (EGE), adult grasshopper extract (AGE), early grasshopper hydrolysate (EGH), and adult grasshopper hydrolysate (AGH).

Phenolic subgroup	Phenolic compounds	Samples			
		EGE (μg/*g*)	AGE (μg/*g*)	EGH (μg/*g*)	AGH (μg/*g*)
Phenolic acids	Protocatechuic acid	114.03^a^ + 5.91	88.44^b^ + 9.95	7.88^c^ + 0.21	19.09^c^ + 0.89
	4-hydroxybenzoic acid	82.15^a^ + 2.76	14.58^b^ + 0.01	14.20^b^ + 0.18	12.42^b^ + 0.74
	4-hydroxyphenylacetic acid	4.20^b^ + 0.67	4.16^b^ + 0.09	10.73^a^ + 0.69	0.79^c^ + 0.10
	4-coumaric acid	2.29^b,c^ + 0.79	27.58^a^ + 3.92	5.96^b^ + 0.06	0.25^c^ + 0.01*
	Vanillic acid	2.06^c^ + 0.19	60.1^a^ + 0.13	0.75^d^ + 0.03	5.55^b^ + 0.29
	Caffeic acid	1.77^b^ + 0.14	7.46^a^ + 0.05	1.69^b^ + 0.03	0.52^c^ + 0.25
	Ferulic acid	1.31^c^ + 0.03	16.81^a^ + 0.14	0.87^d^ + 0.02	2.24^b^ + 0.12
	Salicylic acid	0.52^c^ + 0.08	4.91^b^ + 0.01	0.10^d^ + 0.01	5.51^a^ + 0.35
	Gentisic acid	0.33^b^ + 0.01*	12.99^a^ + 0.05	—	12.87^a^ + 0.76
	Sinapic acid	0.31^c^ + 0.09*	5.17^a^ + 0.08	0.35^c^ + 0.02*	0.69^b^ + 0.03
	*t*-cinnamic acid	0.11^b^ + 0.01*	0.09^c^ + 0.00*	0.15^a^ + 0.00*	0.01^d^ + 0.01*
Phenolic aldehyde	Vanillin	0.14^b^ + 0.03*	0.97^a^ + 0.08	0.10^b^ + 0.01*	0.68^a^ + 0.28*
Coumarin	Umbelliferone	—	0.83^a^ + 0.01	—	0.41^b^ + 0.03
Flavonoids	Luteolin	14.42^a^ + 1.82	8.15^b^ + 0.05	9.41^b^ + 0.21	6.09^c^ + 0.29
	Apigenin	10.72^b^ + 0.91	18.83^a^ + 0.08	5.36^c^ + 0.11	4.69^c^ + 0.24
	Quercetin	3.95^a^ + 2.54	1.36^a^ + 0.02	2.31^a^ + 0.08	1.74^a^ + 0.09
	Kaempferol	1.44^a^ + 0.93	2.00^a^ + 0.04	1.12^a^ + 0.03	—
	Quercetin-3-glucoside	0.83^b^ + 0.79*	13.56^a^ + 0.21	0.35^b^ + 0.01*	—
	Kaempferol-3-O-glucoside	0.41^b^ + 0.22*	5.44^a^ + 0.10	0.15^b^ + 0.01	—
Lignan	Secoisolariciresinol	2.10^c^ + 0.30	24.61^b^ + 0.70	1.01^c^ + 0.19	31.14^a^ + 1.30

Concentration is expressed in microgram/gram (μg/*g*) of dried sample, and it is shown as the average of four determinations plus and minus the standard deviation. “—,” not identified.

*Value determined below the limit of quantification. ^a,b,c,d^Different letters between columns indicate a statistically significant difference based on Tukey *post-hoc* analyses.

Even though a higher content of total phenolic compounds (Figure 1) was obtained in hydrolysates (EGH 195 ± 13.6 mg GAE, AGH 143.5 ± 16.7 mg GAE), according to the mass analysis, phenolic compounds in hydrolysates were lower (EGH 62.49 μg/*g*, AGH 104.69 μg/*g*) than those in EGE and AGE (243.09 μg/*g* and 318.04 μg/*g*, respectively). Hence, the previously reported antioxidant capacity observed in hydrolysates and fractions 3 of hydrolysates is not completely attributed to polyphenols, indicating that other compounds such as peptides could be responsible for this activity. Similarly, after *in vitro* protein digestion, the reducing capacity showed a dramatic decrease (Figure 5), indicating that this antioxidant capacity can be attributed to the presence of peptides that were digested. There are reports of peptide sequences with selective bioactivity. For instance, in a complex peptide mixture from common bean protein hydrolysates inhibiting HCT116 colon cancer cells, treatments with the major synthetic pure peptides identified in the hydrolysates exerted different anticancer effects among them. While some peptides inhibited human colorectal cancer cells by potential DNA damage, other peptides that did not inhibit cancer cell growth had a potent antioxidant activity protecting normal cells from oxidative stress (56).

## Conclusion

*Sphenarium purpurascens* represents an alternative source of food ingredients with a high nutritional value. In this work, the use of grasshoppers as a source of antioxidant compounds is evidenced. Particularly, hydrolysates from grasshoppers presented high antioxidant capacity. Further identification and characterization of compounds other than phenolics such as peptides that could be responsible for antioxidant capacity is necessary for exploring their potential uses. In addition, it is important to mention the relevance of the bioaccessibility and bioavailability analysis of bioactive compounds to corroborate their functionality after digestion. In this case, it is necessary to develop strategies to preserve the biological activity of compounds in extracts and hydrolysates since some of these compounds can be degraded after digestion. For instance, the use of a food matrix that serves as a vehicle to maintain the bioaccessibility of bioactive compounds in grasshopper extracts and hydrolysates can be evaluated.

## Data availability statement

The original contributions presented in this study are included in the article/Supplementary material, further inquiries can be directed to the corresponding author.

## Author contributions

MM-M was responsible for the experimentation, data analyses, investigation, and writing of the manuscript. JM-V and JG-A were responsible for UPLC-MS and data analysis. CI-H designed the experimental strategy and responsible for the project. MM-M and DL-V contributed to the design of the experimental strategy. DL-V and CI-H reviewed the manuscript. All authors reviewed the final version of the manuscript.
